# Supplementary material for: The divergence history of European blue mussel species reconstructed from Approximate Bayesian Computation: the effects of sequencing techniques and sampling strategies
Source: PeerJ. 2018 Jul 30;6:e5198. doi: 10.7717/peerj.5198 (PMC6071616; doi:10.7717/peerj.5198)
Supplement: Supplemental Information 1 [file peerj-06-5198-s001.pdf]

Table S1. Posterior probabilities of the 11 models in the mussel datasets

technique	n1	n2	statistics	PP										
				SI	IM.hetero	AM.hetero	SC.hetero	PAM.hetero	PSC.hetero	IM.homo	AM.homo	SC.homo	PAM.homo	PSC.homo
exome capture	2	2	s4	0.028	0.002	0.159	0.002	0.249	0.001	0.001	0.193	0.002	0.360	0.003
exome capture	2	2	s7	0.060	0.006	0.139	0.004	0.246	0.003	0.001	0.230	0.001	0.309	0.001
exome capture	2	2	s23	0.009	0.251	0.007	0.319	0.007	0.385	0.002	0.006	0.004	0.007	0.003
exome capture	2	2	mscalc	0.369	0.002	0.235	0.094	0.050	0.031	0.000	0.147	0.033	0.023	0.016
exome capture	4	4	s4	0.023	0.019	0.126	0.014	0.255	0.013	0.004	0.200	0.003	0.340	0.003
exome capture	4	4	s7	0.015	0.050	0.207	0.030	0.213	0.050	0.003	0.168	0.006	0.250	0.009
exome capture	4	4	s23	0.000	0.332	0.000	0.228	0.001	0.363	0.004	0.000	0.049	0.001	0.022
exome capture	4	4	mscalc	0.006	0.078	0.020	0.396	0.051	0.342	0.009	0.006	0.022	0.017	0.054
exome capture	8	8	s4	0.053	0.005	0.151	0.004	0.293	0.004	0.001	0.155	0.001	0.334	0.001
exome capture	8	8	s7	0.074	0.007	0.157	0.007	0.254	0.011	0.001	0.151	0.001	0.337	0.002
exome capture	8	8	s23	0.000	0.132	0.000	0.243	0.000	0.609	0.005	0.000	0.007	0.000	0.004
exome capture	8	8	mscalc	0.344	0.001	0.002	0.119	0.001	0.232	0.000	0.003	0.074	0.000	0.223
rna-seq	2	2	s4	0.004	0.056	0.142	0.099	0.227	0.090	0.016	0.137	0.017	0.200	0.011
rna-seq	2	2	s7	0.072	0.098	0.200	0.092	0.323	0.036	0.000	0.037	0.000	0.142	0.001
rna-seq	2	2	s23	0.009	0.159	0.005	0.388	0.003	0.414	0.000	0.003	0.010	0.007	0.001
rna-seq	2	2	mscalc	0.005	0.113	0.060	0.312	0.071	0.344	0.000	0.001	0.071	0.004	0.018
rna-seq	4	4	s4	0.026	0.127	0.097	0.167	0.180	0.132	0.000	0.095	0.008	0.158	0.009
rna-seq	4	4	s7	0.034	0.017	0.155	0.019	0.339	0.034	0.001	0.158	0.003	0.237	0.004
rna-seq	4	4	s23	0.017	0.252	0.027	0.236	0.042	0.346	0.000	0.028	0.003	0.039	0.009
rna-seq	4	4	mscalc	0.011	0.168	0.045	0.164	0.072	0.420	0.001	0.039	0.007	0.060	0.015

technique: rna sequencing ("rna-seq") vs. exome enrichment sequencing ("exome capture"); **n**: number of individuals analyzed in each species;
statistics: *jsfs*=4 (4 classes), *jsfs*=7 (7 classes), *jsfs*=23 (23 classes), mscalc (28 summary statistics); **PP**: posterior probability;
11 models: SI, IM hetero, IM homo, AM homo, AM hetero, PAM homo, PAM hetero, SC homo, SC hetero, PSC hetero, PSC homo.

Table S2. Posterior probabilities of the homo *vs.* hetero models in the mussel datasets

technique	n1	n2	statistics	IM best_model	IM PP	AM best_model	AM PP	PAM best_model	PAM PP	SC best_model	SC PP	PSC best_model	PSC PP
exome capture	2	2	jsfs=4	hetero	0.656	homo	0.542	homo	0.557	hetero	0.592	hetero	0.527
exome capture	2	2	jsfs=7	hetero	0.860	homo	0.578	homo	0.695	hetero	0.734	hetero	0.771
exome capture	2	2	jsfs=23	hetero	0.991	homo	0.530	homo	0.632	hetero	0.893	hetero	0.990
exome capture	2	2	mscalc	hetero	0.960	hetero	0.827	hetero	0.712	hetero	0.938	hetero	0.823
exome capture	4	4	jsfs=4	hetero	0.814	homo	0.512	homo	0.538	hetero	0.898	hetero	0.794
exome capture	4	4	jsfs=7	hetero	0.839	homo	0.539	homo	0.519	hetero	0.958	hetero	0.956
exome capture	4	4	jsfs=23	hetero	0.999	hetero	0.722	hetero	0.816	hetero	0.916	hetero	0.982
exome capture	4	4	mscalc	hetero	0.971	hetero	0.703	homo	0.509	hetero	0.955	hetero	0.916
exome capture	8	8	jsfs=4	hetero	0.854	hetero	0.580	homo	0.509	hetero	0.892	hetero	0.769
exome capture	8	8	jsfs=7	hetero	0.901	hetero	0.576	homo	0.551	hetero	0.969	hetero	0.905
exome capture	8	8	jsfs=23	hetero	0.953	hetero	0.618	hetero	0.675	hetero	0.926	hetero	1.000
exome capture	8	8	mscalc	hetero	0.794	homo	0.643	hetero	0.771	hetero	0.933	hetero	0.807
rna-seq	2	2	jsfs=4	hetero	0.970	homo	0.519	hetero	0.556	hetero	0.837	hetero	0.832
rna-seq	2	2	jsfs=7	hetero	0.916	homo	0.523	hetero	0.733	hetero	0.973	hetero	0.946
rna-seq	2	2	jsfs=23	hetero	0.864	homo	0.662	hetero	0.671	hetero	0.948	hetero	0.818
rna-seq	2	2	mscalc	hetero	0.983	hetero	0.595	homo	0.687	hetero	0.969	hetero	0.987
rna-seq	4	4	jsfs=4	hetero	0.975	hetero	0.511	hetero	0.575	hetero	0.895	hetero	0.893
rna-seq	4	4	jsfs=7	hetero	0.996	homo	0.526	homo	0.520	hetero	0.956	hetero	0.929
rna-seq	4	4	jsfs=23	hetero	1.000	hetero	0.780	hetero	0.824	hetero	0.999	hetero	0.997
rna-seq	4	4	mscalc	hetero	0.993	homo	0.584	hetero	0.583	hetero	1.000	hetero	0.986

technique: rna sequencing ("rna-seq") vs. exome enrichment sequencing ("exome capture"); **n**: number of individuals analyzed in each species; **statistics**: *jsfs*=4 (4 classes), *jsfs*=7 (7 classes), *jsfs*=23 (23 classes), mscalc (28 summary statistics); **PP**: posterior probability.

Table S3. Accuracy and ambiguity rate in the simulated datasets

statistics	target_model	(A) homo *vs.* hetero accuracy_rate	(A) homo *vs.* hetero ambiguity_rate	(B) 6 models (hetero) accuracy_rate	(B) 6 models (hetero) ambiguity_rate
jsfs=4	IM hetero	-	1	-	0.99
jsfs=7	IM hetero	1	0.98	0.70	0.93
jsfs=23	IM hetero	1	0.34	1.00	0.25
mscalc	IM hetero	1	0.38	0.97	0.4
jsfs=4	AM hetero	-	1	0.34	0.47
jsfs=7	AM hetero	-	1	0.32	0.48
jsfs=23	AM hetero	1	0.57	0.42	0.26
mscalc	AM hetero	1	0.7	0.44	0.21
jsfs=4	PAM hetero	-	1	0.65	0.45
jsfs=7	PAM hetero	-	1	0.70	0.41
jsfs=23	PAM hetero	1	0.69	0.79	0.2
mscalc	PAM hetero	1	0.63	0.86	0.14
jsfs=4	SC hetero	-	1	0.57	0.88
jsfs=7	SC hetero	1	0.97	0.48	0.86
jsfs=23	SC hetero	1	0.3	0.50	0.31
mscalc	SC hetero	1	0.46	0.43	0.61
jsfs=4	PSC hetero	-	1	0.75	0.92
jsfs=7	PSC hetero	1	0.99	0.89	0.86
jsfs=23	PSC hetero	1	0.37	1.00	0.3
mscalc	PSC hetero	1	0.33	1.00	0.52
jsfs=4	SI	-	-	0.71	0.27
jsfs=7	SI	-	-	0.73	0.21
jsfs=23	SI	-	-	0.85	0.15
mscalc	SI	-	-	0.92	0.13

Cross validations have been performed on simulated datasets that mimic the feature of the "exome capture" data with *n*=2 individuals. 100 pseudo-observed datasets were produced for each tested model, and a minimal posterior probability threshold, P_{min}, was set-up to be 1/3 higher than the prior expectation, i.e. 1/2+1/3=0.83 in "homo vs. hetero", and 1/6+1/3=0.43 in "6 models (hetero)".

statistics: *jsfs*=4 (4 classes), *jsfs*=7 (7 classes), *jsfs*=23 (23 classes), mscalc (28 summary statistics);
accuracy_rate: proportion of simulated datasets that correctly supported model M, given a minimal posterior probability (P_{min}). This is the proportion, among simulated datasets generated under all tested models and inferred by ABC to correspond to model M, of those actually generated under model M;
ambiguity_rate: proportion of simulated datasets generated under model M whose posterior probability is below a certain threshold (Pmin), such that model choice is ambiguous;
6 models (hetero): SI, IM hetero, AM hetero, PAM hetero, SC hetero, PSC hetero.
