# Supplementary material for: The divergence history of European blue mussel species reconstructed from Approximate Bayesian Computation: the effects of sequencing techniques and sampling strategies
Source: PeerJ. 2018 Jul 30;6:e5198. doi: 10.7717/peerj.5198 (PMC6071616; doi:10.7717/peerj.5198)
Supplement: Supplemental Information 2 [file peerj-06-5198-s002.pdf]

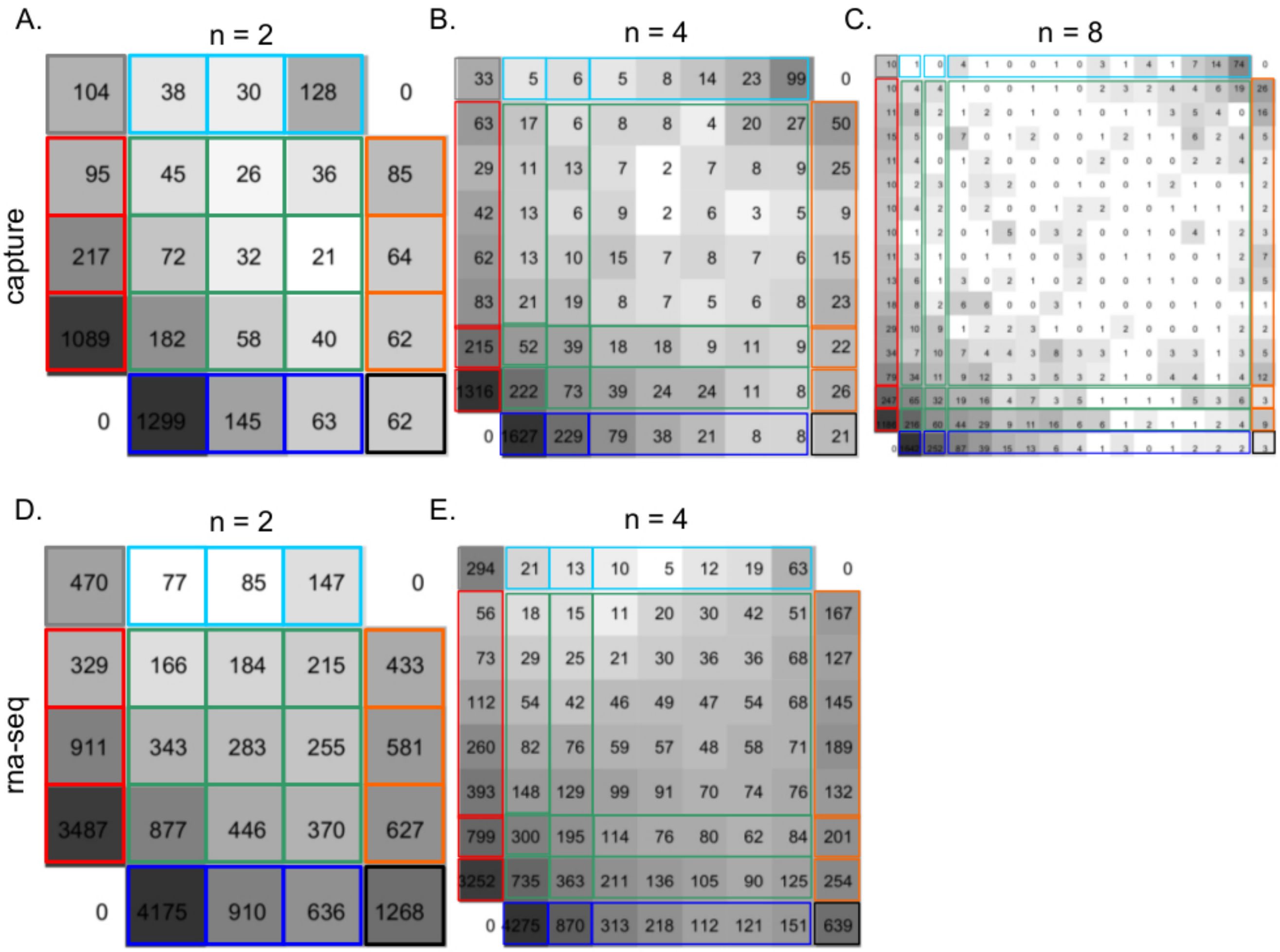

Figure S1. Unfolded joint site frequency spectrum for the two different sequencing techniques. jSFS of increasing sample size are shown for the “exome capture” data sets in (A) two individuals: 3,993 SNPs, (B) four individuals: 5,092 SNPs and (C) eight individuals: 5,000 SNPs; and for the “rna-seq” data sets in (D) two individuals: 17,275 SNPs and (E) four individuals: 17,902 SNPs. For $n < 8$, individuals were subsampled at random. Each cell is colored proportionally to the logarithm of the number of sites within it (indicated inside each cell). The decomposition into 23 classes of polymorphism is shown. All other details match Figure 2.

A. jsfs = 23 *PSC.hetero (best model; PP=0.385)*

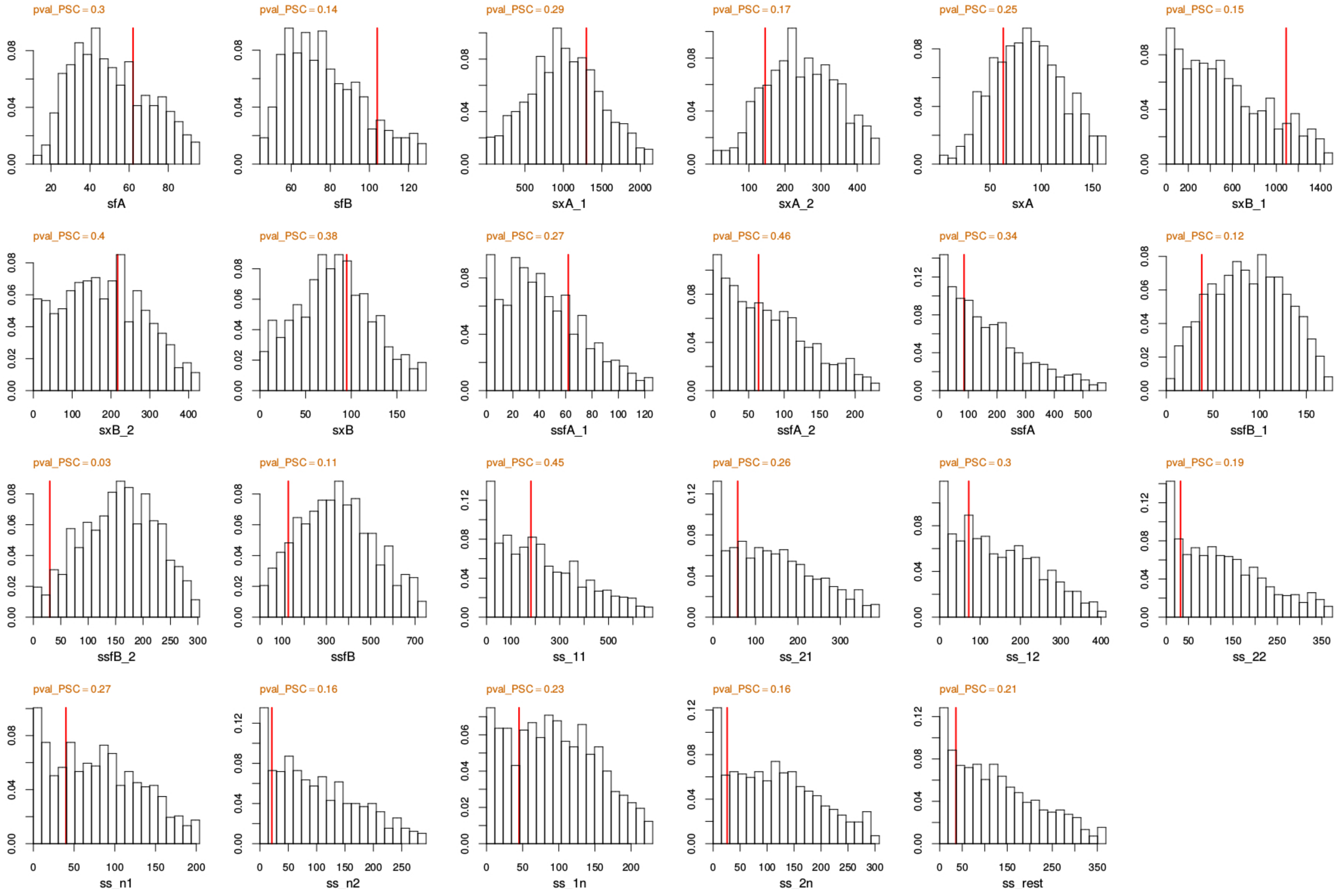

PAM.hetero (6th model; PP=0.007)

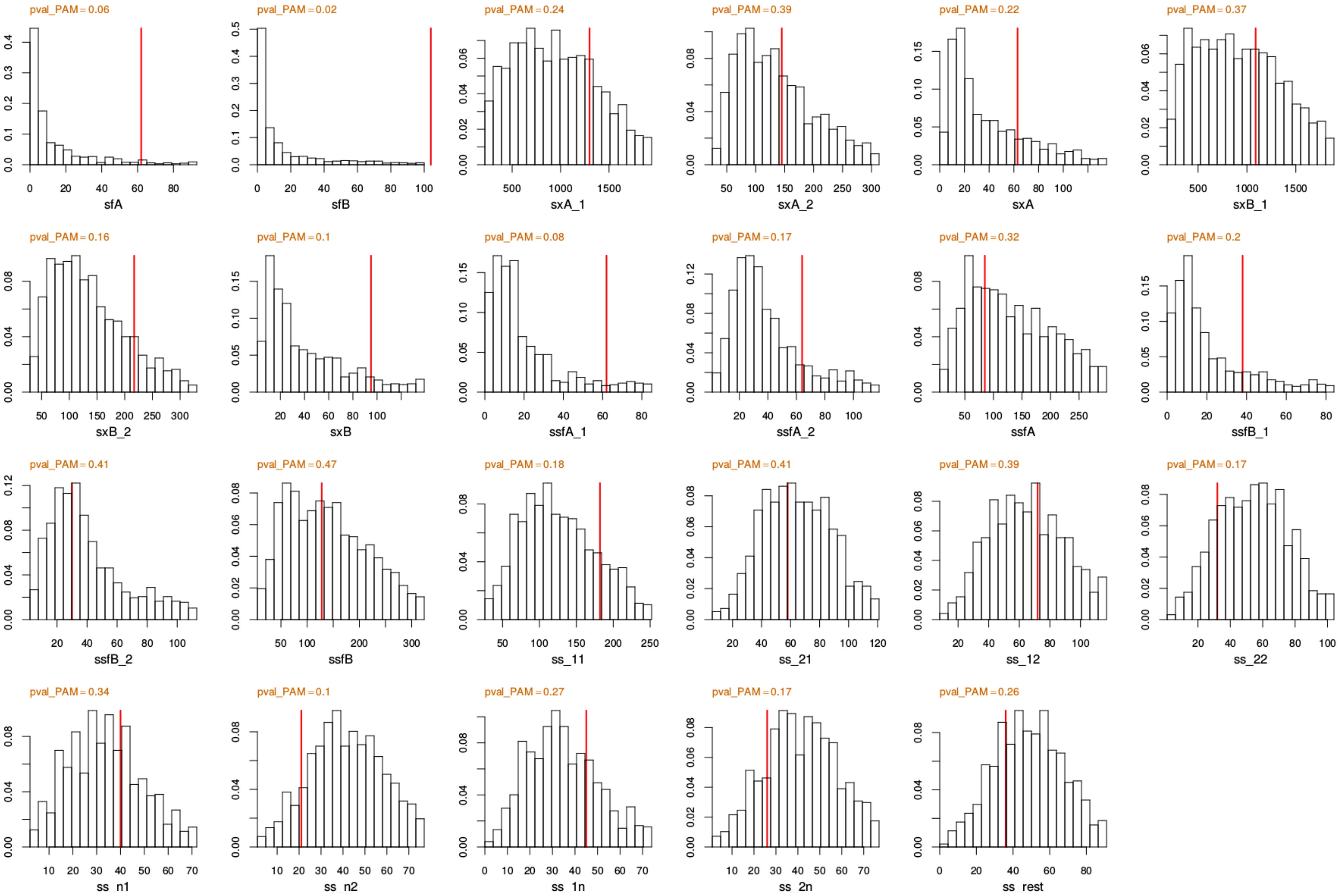

SI (4th model; PP=0.009)

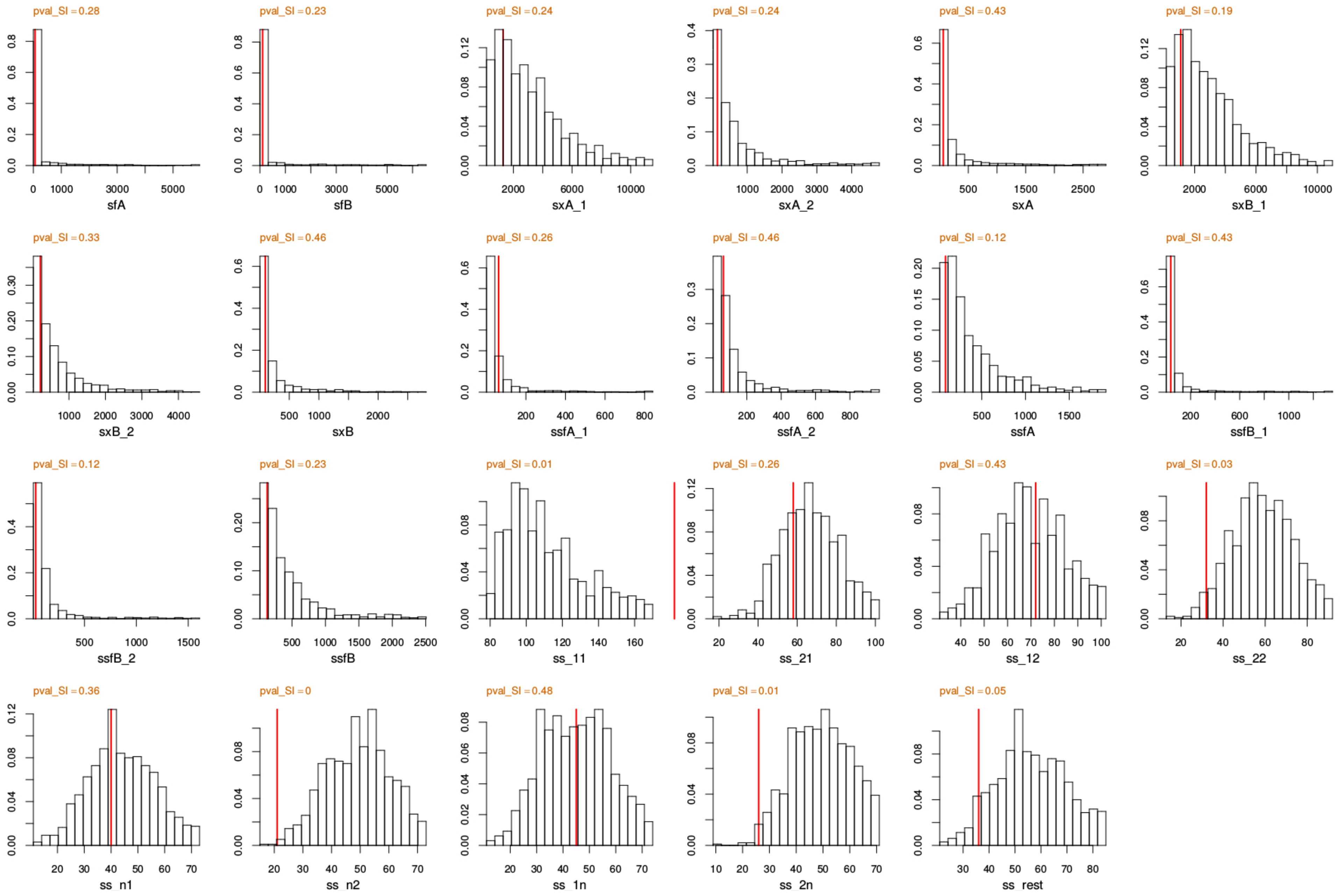

B. jsfs = 7

PSC.homo (9th model; PP=0.001)

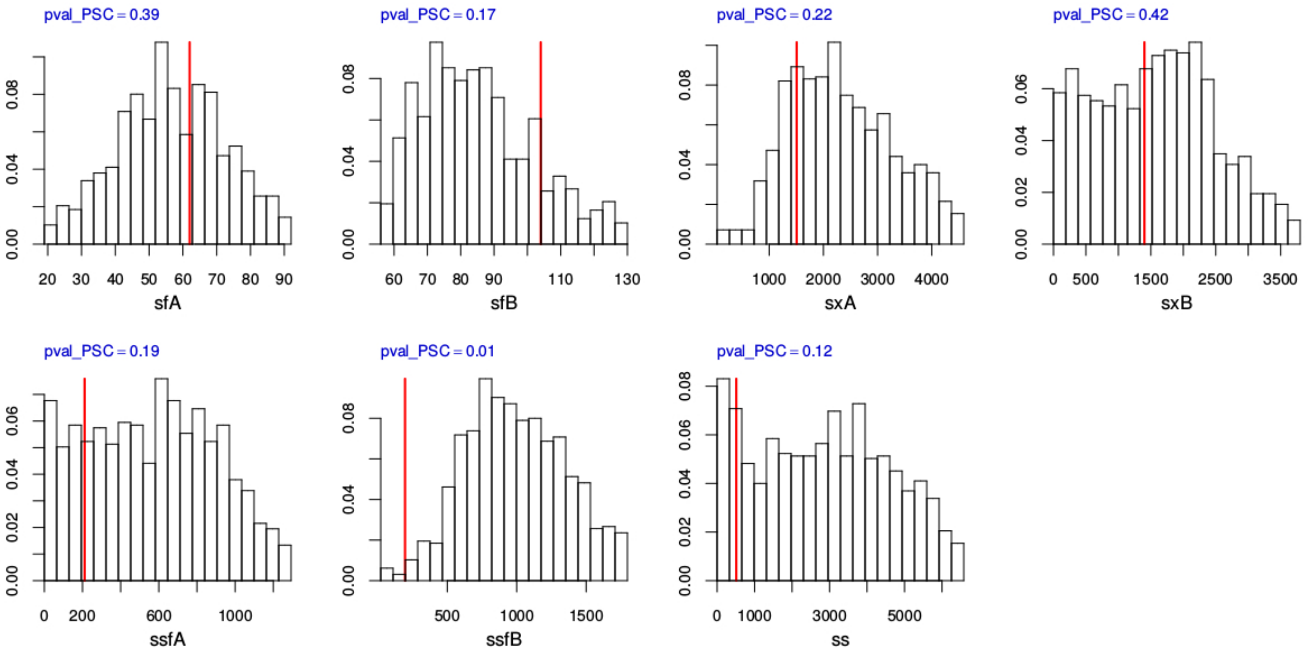

PAM.homo (best model; PP=0.309)

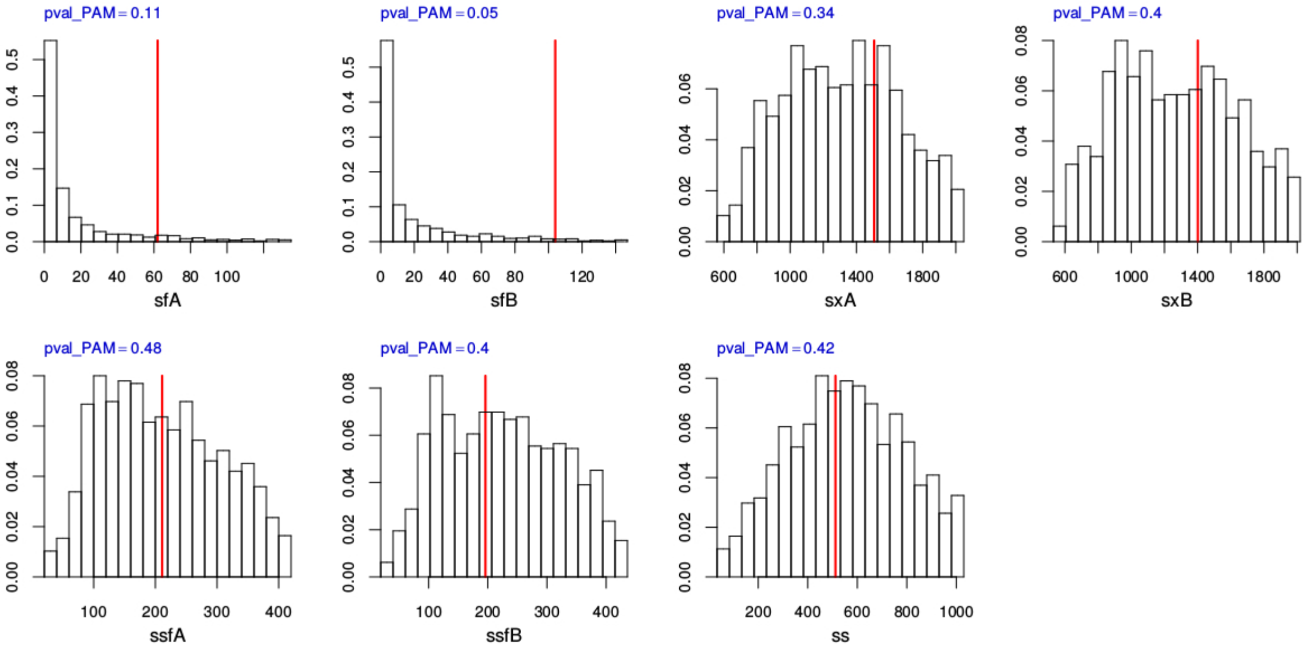

SI (5th model; PP=0.06)

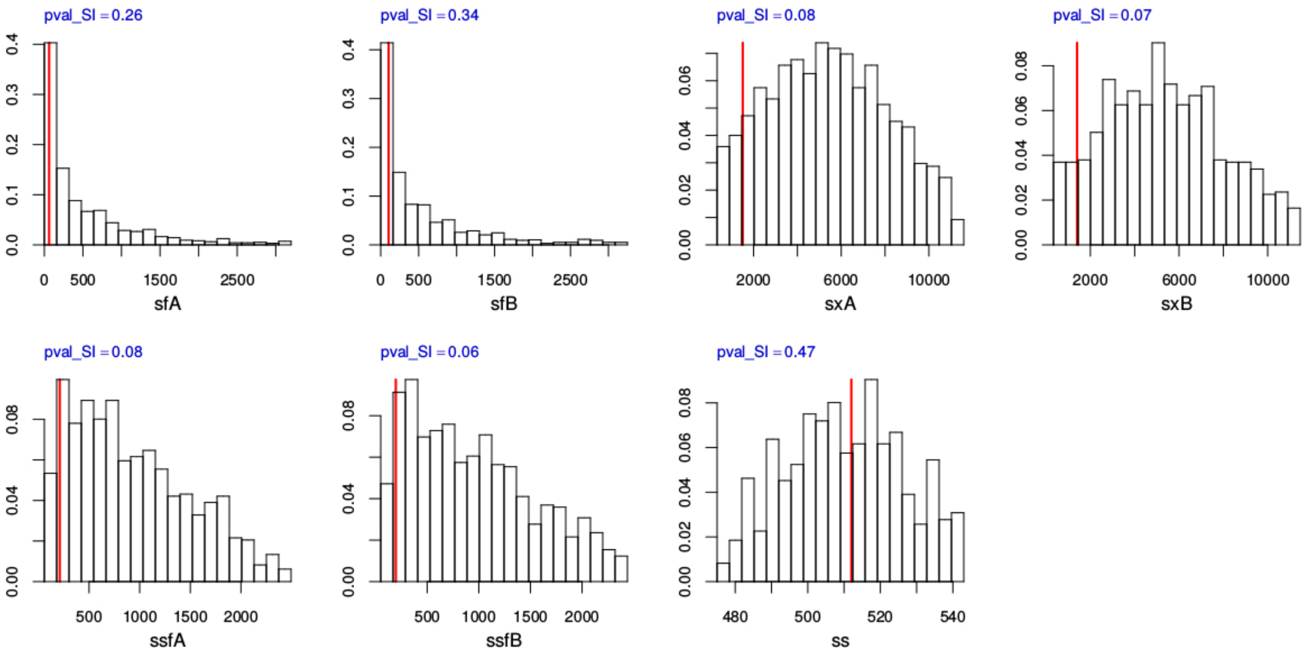

C. jsfs = 4

PSC.homo (6th model; PP=0.003)

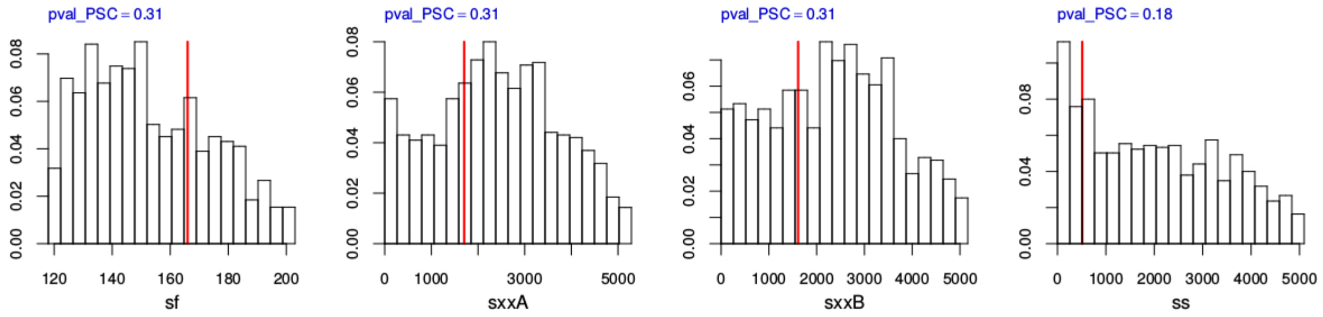

PAM.homo (best model; PP=0.360)

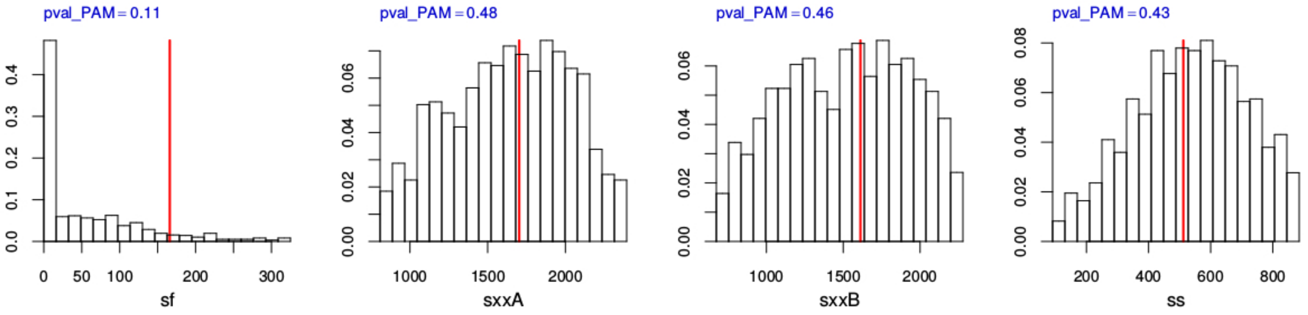

SI (5th model; PP=0.028)

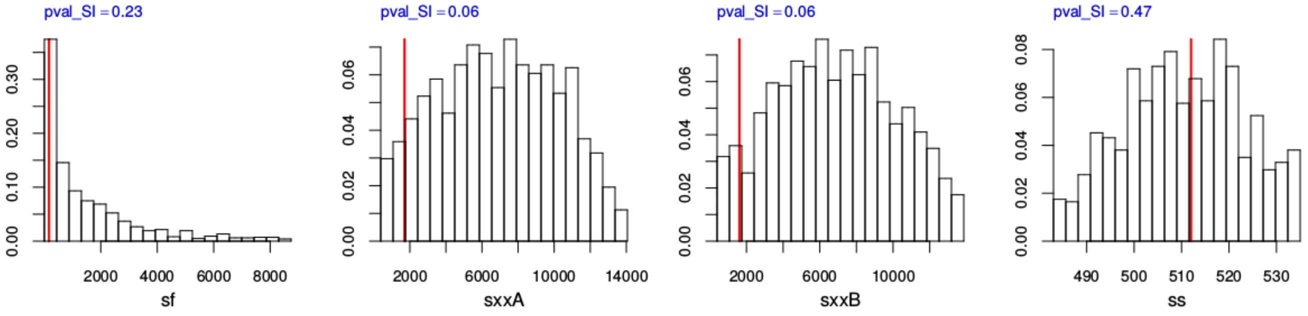

D. mscalc

PSC.hetero

(7th model; PP=0.031)

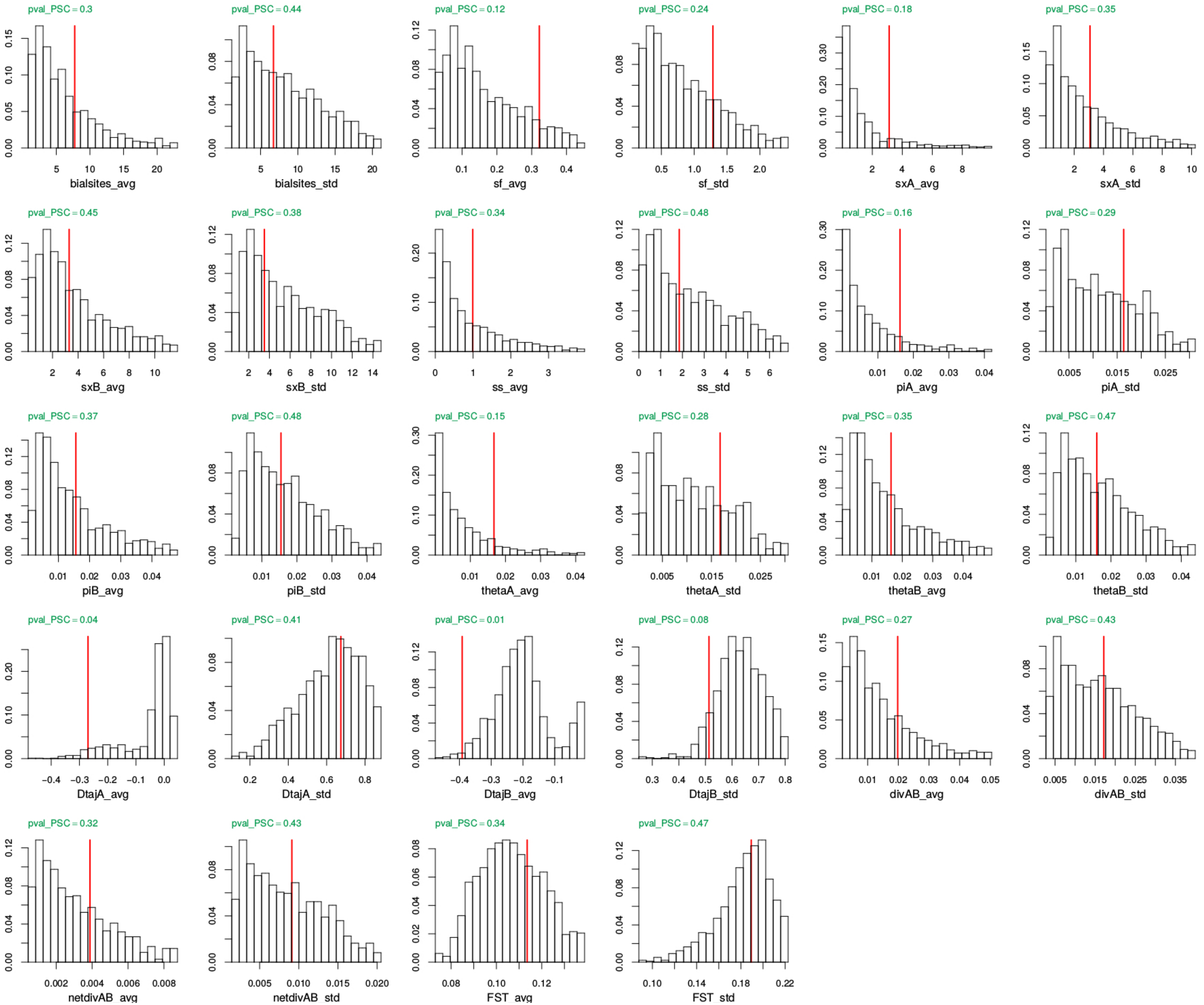

PAM.hetero

(5th model; PP=0.05)

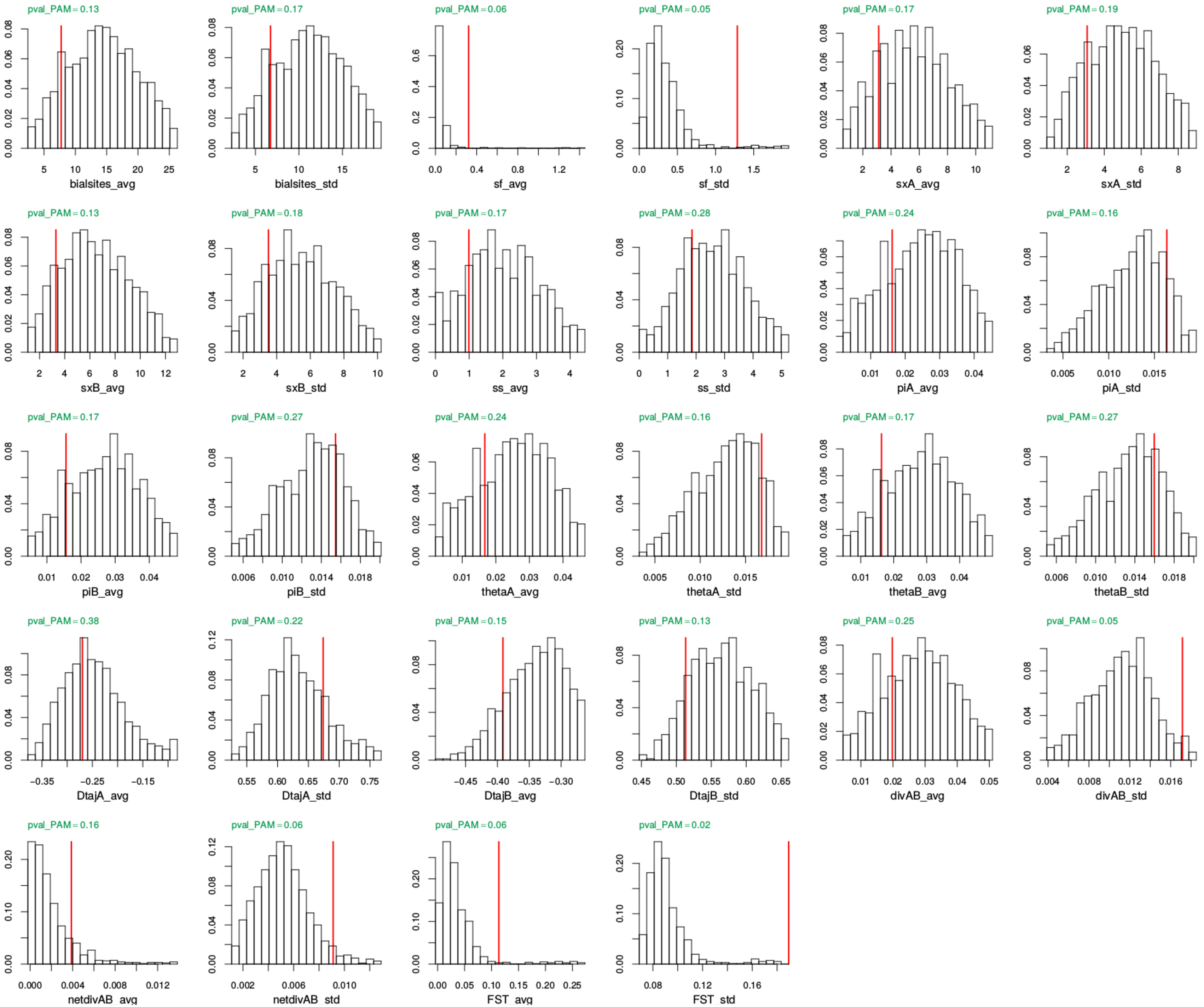

SI (best model; PP=0.369)

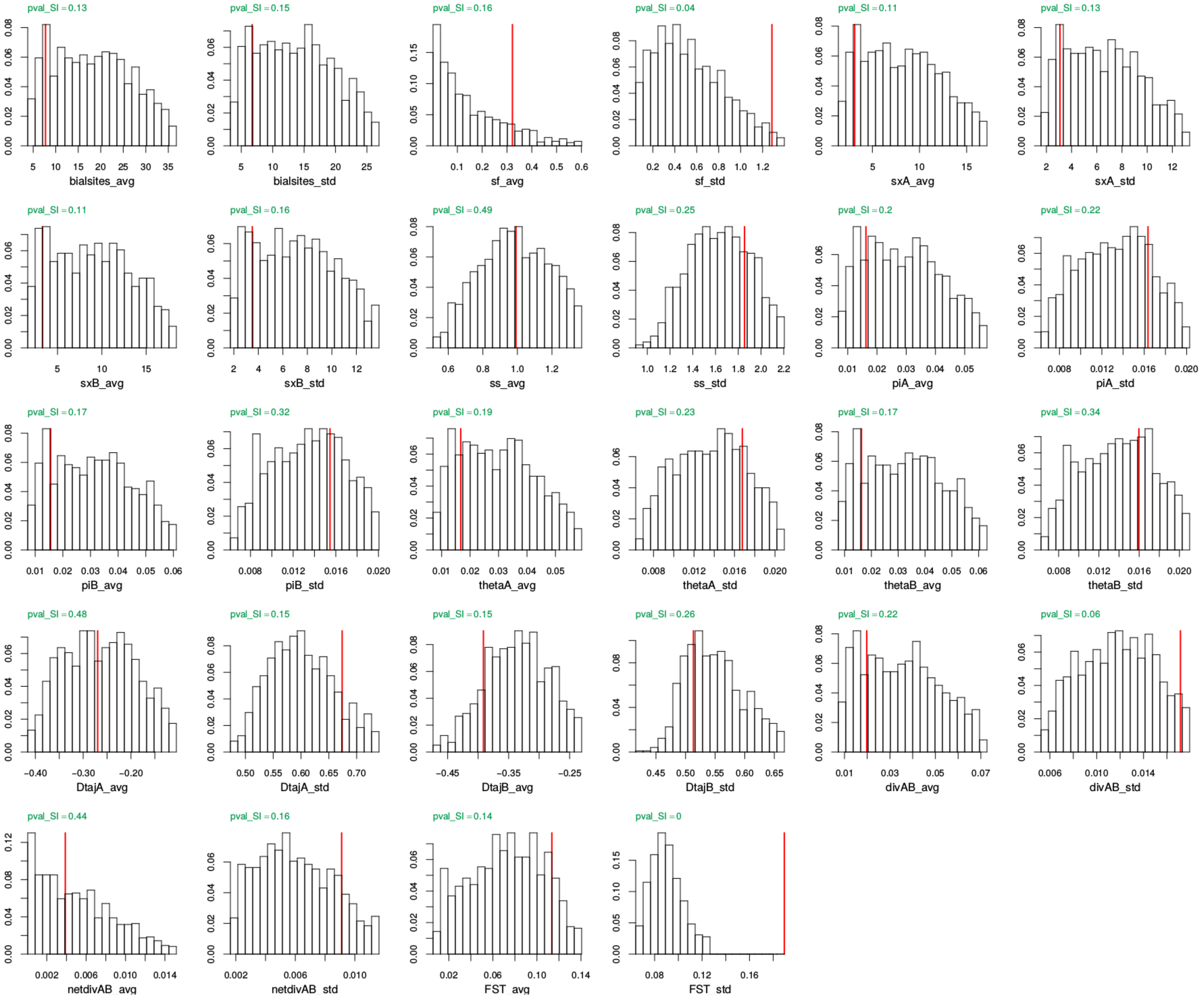

Figure S2. Goodness-of-fit test for three models (Periodic Secondary Contact, PSC; Periodic Ancient Migration, PAM; Strict Isolation, SI) with the "exome capture" data, *n*=2 individuals and the different summary statistics: (A) jSFS including twenty-three classes (best model: PSC); (B) jSFS including 7 classes (best model: PAM); (C) jSFS including 4 classes (best model: PAM); (D) mscalc (best model: SI). 100 multilocus pseudo-observed datasets were simulated from the joint posterior distribution of parameters under each best-estimated model. From these simulations, we compared the expected distributions of the summary statistics with their observed value. For each statistics, the red vertical line represents the observed value, and the bars represent the posterior distribution.

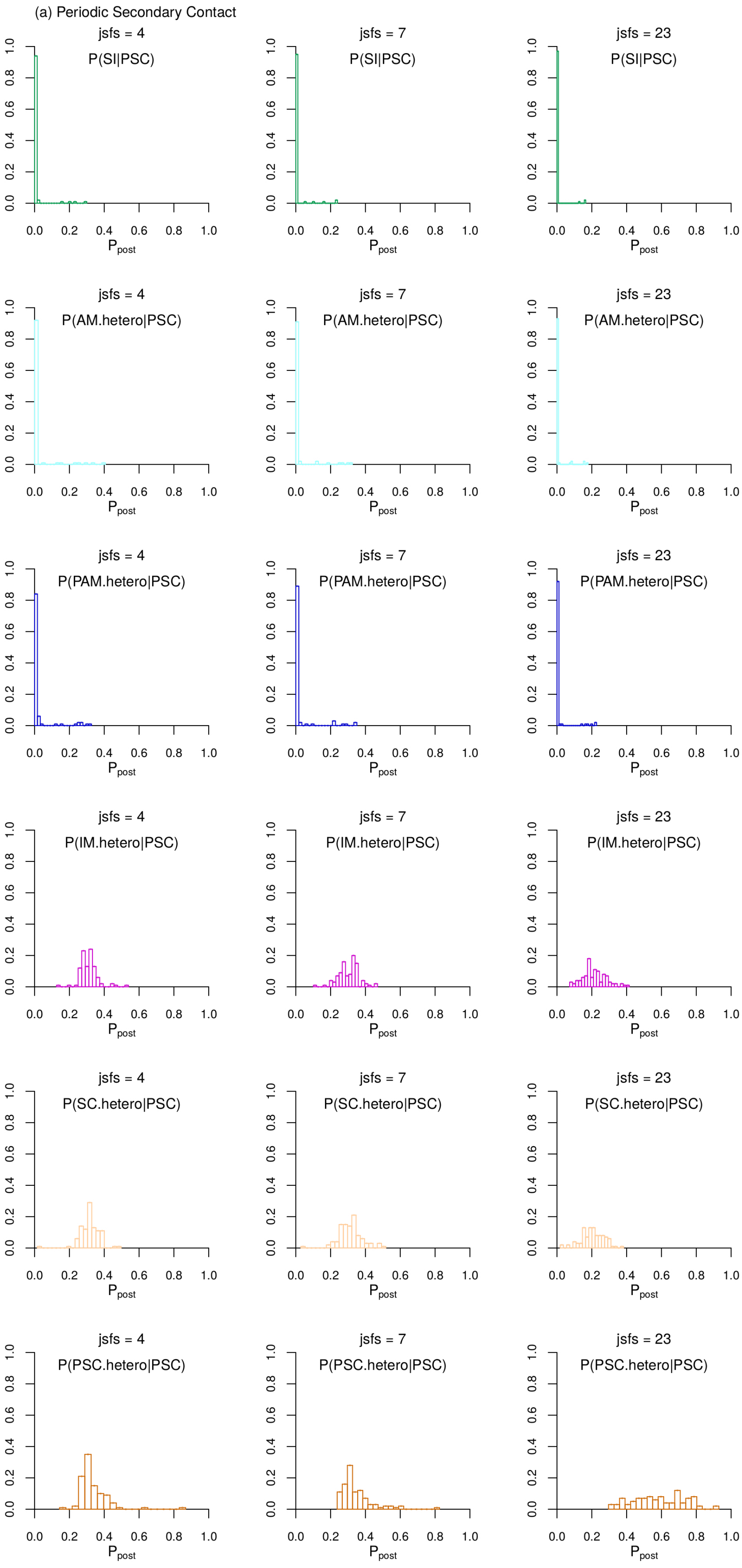
(a) Periodic Secondary Contact
jsfs = 4
jsfs = 7
jsfs = 23
P(SI|PSC)
P(AM.hetero|PSC)
P(PAM.hetero|PSC)
P(IM.hetero|PSC)
P(SC.hetero|PSC)
P(PSC.hetero|PSC)
P_{post}
0.0 0.2 0.4 0.6 0.8 1.0

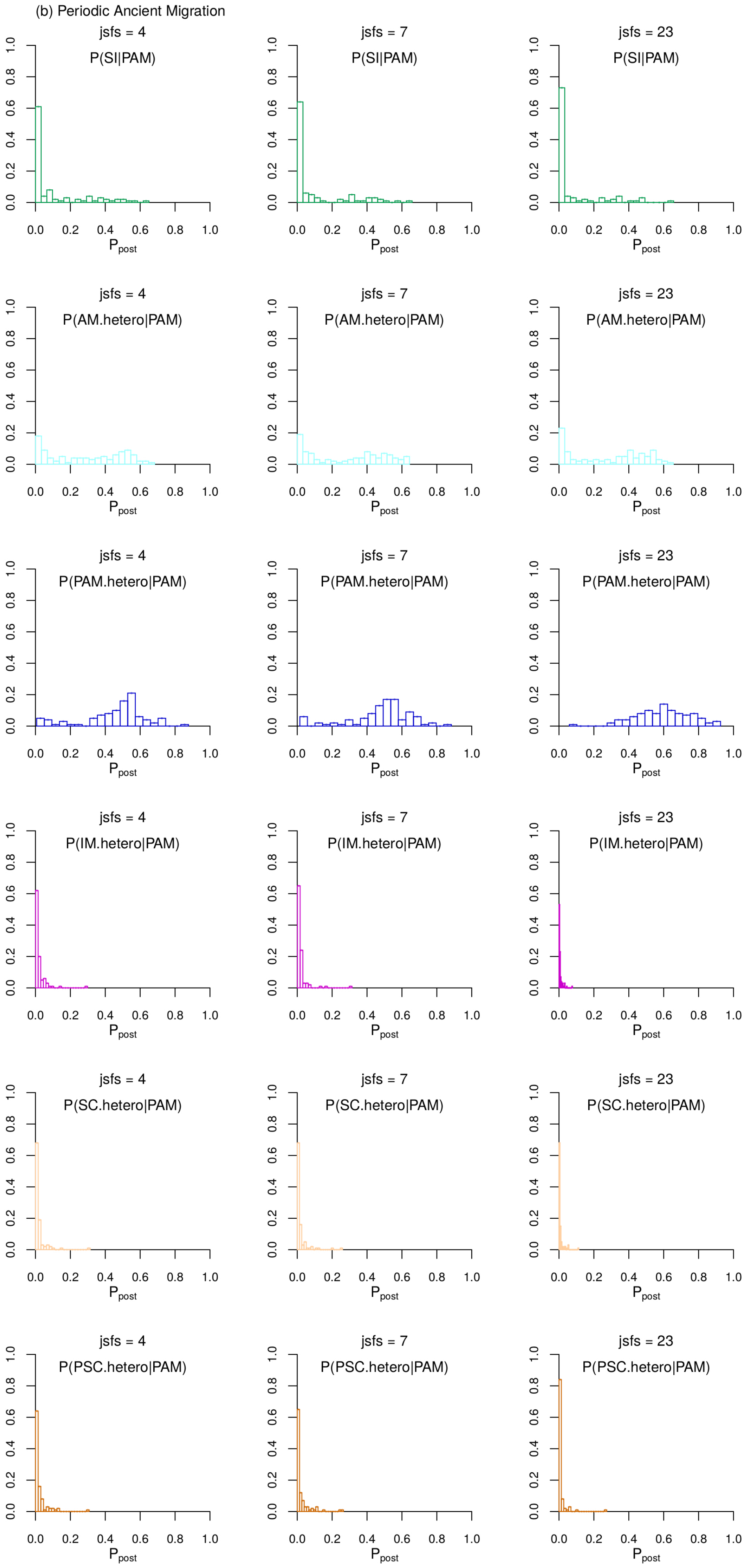
(b) Periodic Ancient Migration
jsfs = 4
jsfs = 7
jsfs = 23
P(SI|PAM)
P(AM.hetero|PAM)
P(PAM.hetero|PAM)
P(IM.hetero|PAM)
P(SC.hetero|PAM)
P(PSC.hetero|PAM)
0.0
0.2
0.4
0.6
0.8
1.0
P_{post}

Figure S3. Evaluation of the ABC performance on model choice. Empirical distributions of the posterior probability of (A) the periodic secondary contact model and (B) the periodic ancient migration model are shown when Strict Isolation (green), Isolation with Migration (purple), Ancient Migration (pale blue), Periodic Ancient Migration (blue), Secondary Contact (pale orange) or Periodic Seconday Contact (orange) models are the true models. Distributions were obtained from an ABC analysis of 100 simulated pseudo-observed datasets. Results for the different binning strategies (*jsfs*=4, *jsfs*=7 and *jsfs*=23 classes) are compared.
